# Evaluation of Passive Films on 17-7PH and 410 Stainless Steel Exposed to NaCl Solution

**DOI:** 10.3390/ma17164060

**Published:** 2024-08-15

**Authors:** Brisa Martínez-Aparicio, Citlalli Gaona-Tiburcio, Facundo Almeraya-Calderon, Reece Goldsberry, Homero Castaneda

**Affiliations:** 1 Universidad Autónoma de Nuevo León, FIME, Centro de Investigación e Innovación en Ingeniería Aeronáutica (CIIIA), San Nicolás de los Garza 66455, Mexicofacundo.almerayacld@uanl.edu.mx (F.A.-C.); 2 Department of Materials Science & Engineering, Texas A&M University, 209 Reed McDonald Building, College Station, TX 77840, USA; reece1579@tamu.edu

**Keywords:** corrosion protection, passivation, 17-7PH, 410 SS, passive film, XPS

## Abstract

This work covers the formation of a passive state for two different alloys used in the aeronautical industry. The aim of this study is to investigate the effectiveness of passivation treatments on 17-7PH and 410 SS (stainless steel) samples, specifically when performed with citric and nitric acid solutions at 49 °C using an immersion time of 90 min and subsequent exposure in 3.5 wt.% NaCl solution. Employing the cyclic potentiodynamic polarization (CPP) technique, the corrosion properties of the passivated material were evaluated according to the ASTM G65-11 standard. A microstructural analysis was performed using scanning electron microscopy (SEM). The passivated layer was characterized via X-ray photoelectron spectroscopy. In the results, the CPP curves showed positive hysteresis, indicating pitting localized corrosion, and 17-7PH steel passivated at 49 °C for 90 min in citric acid exhibited lower corrosion rate values equivalent to ×10^−3^ mm/year.

## 1. Introduction

In multiple sectors, corrosion can lead to significant economic losses. The associated costs of corrosion can vary considerably depending on factors such as industry regulations, technological advancements, and preventive maintenance practices [1,2]. This is pertinent in the case of aircraft and aerospace equipment [3,4], where corrosion due to exposure to chemical substances and atmospheric conditions can incur substantial maintenance, repair, and inspection costs. Numerous methods are available for preventing corrosion, including coating application, surface treatments, and the appropriate selection of materials. In the aeronautical sector, composite materials, titanium, aluminum alloys and steels are some of the most used materials [5] due to the high requirements of material selection. Steel is employed in smaller amounts than the other material groups; however, is commonly used in critical safety/structural components.

In the aeronautical industry, stainless steels are commonly employed due to their inherently high corrosion resistance and mechanical properties. Among the classifications of stainless steels, one of the commonly used classes is precipitation-hardenable stainless steels (PHSS) [6]. These alloys primarily consist of austenite at specific temperatures, and their ability to retain austenite after cooling is essential to provide important mechanical properties or form specific microstructures. They are categorized into three types: martensitic (α′), austenitic (γ), and semi-austenitic (γ and δ) [7]. Regarding precipitation-hardenable semi-austenitic stainless steels, such as alloy 17-7PH, a transformation to martensite before precipitation hardening can occur through mechanical deformation or thermal processes. 17-7PH stainless steel is characterized by a martensitic-tempered microstructure, which significantly improves its mechanical strength [6,7,8]. In the aeronautical sector, various types of stainless steel are used in different components due to their properties. Martensitic stainless steels are used for components that need high strength-to-weight ratios, while austenitic stainless steels are employed for components that require corrosion resistance and ductility. This includes the 17-4PH, 17-7PH, and 15-7Mo stainless steels developed by Armco (Houston, TX, USA) in 1948. Subsequently, PHSS steels AM350 and AM355 (semi-austenitic) and Custom 630, 455, and 450 (martensitic) were produced [8,9]. This group of alloys meets the required criteria for application in aircraft components, including landing gear and wing-root attachments [5].

Combining elements in the chemical composition also plays an important role in providing various characteristics to stainless steel, and each type of element used is different. In general, precipitation-hardenable stainless steels are made by improving a base stainless steel by using specific elements that facilitate the production of precipitates [6,7]. Adding nickel (Ni) benefits ductility, but the main objective is to induce an austenitic microstructure. For PHSS, nickel is also used to create intermetallic compounds (for example, compounds including niobium, tungsten, aluminum, and titanium) that enhance strength. In martensitic steels, the presence of molybdenum (Mo) increases hardness at higher tempering temperatures, which helps to improve localized corrosion resistance [8,9]. Chromium (Cr) is universally recognized as an essential element in stainless-steel alloys. According to ASM International (the American Society for Metals), stainless steels are iron-based alloys that are made up of a minimum of approximately 12% chromium [10]. This chromium content is crucial because it facilitates the formation of a thin and robust oxide layer on the surface of the steel. This layer, rich in chromium oxide (Cr_2_O_3_) and nanometers thick, provides exceptional corrosion resistance [11,12,13]. The formation of passive films on a material’s surface provides resistance to corrosion in humid air and salt water, so it is essential for preserving stainless steel [13].

When the passive layer is defective, damaged, or destroyed, the corresponding metal is susceptible to active corrosion in the areas where the passive film has been damaged. Because corrosive conditions can degrade mechanical properties like fatigue strength and load capacity [14,15,16], protective methods such as passivation are required. The passivation process is used to guarantee that a material reforms rapidly and is compact, adherent, and continuous. Passivation is the formation of a physical natural layer that confers resistance to corrosion in stainless steel. The passivation process is commonly applied to stainless steels through an oxidizing agent, as per the ASTM A967 standard [17]. One of the main purposes of employing passivation treatments for stainless-steel components in the aeronautical industry is to remove any foreign contaminants from the components’ surfaces. Additionally, it is used to form a compact passive film that can protect the components for longer exposure durations than for air-formed passive films. Due to the critical nature of stainless-steel components in the aerospace environment, it is necessary to take all precautions necessary to ensure that these components do not have an increased chance of failure due to corrosion. Currently, there are alternatives to the traditional nitric acid passivation process, such as the use of citric acid because it is environmentally friendly and biodegradable. According to current research, there is limited information available on the benefits of employing citric acid for the passivation of stainless steel [18,19,20,21]. The use of citric acid as an alternative for the passivation of stainless steel in aeronautical components was tested by the Boeing Company in 2003, followed by the National Aeronautics and Space Administration (NASA) in 2008. They reported high effectiveness of this chemical treatment [14,22,23].

Studies on the behavior of stainless steels regarding resistance to corrosion have focused on localized corrosion, pitting nucleation, passive and trans-passive regions, and corrosion mechanisms. Electrochemical techniques such as potentiodynamic polarization (PP), cyclic potentiodynamic polarization (CPP), and electrochemical impedance spectroscopy (EIS) are the most used techniques. Suresh and Mudali [24] studied the corrosion of AISI 304 stainless steel in a ferric chloride (FeCl_3_) solution using the electrochemical noise technique. The findings indicated an important relationship between the frequency domain (power spectral density, PSD) and the time domain (statistical analysis) in regard to determining the localized corrosion mechanism. In 2018, Lara et al. [25] studied the corrosion kinetics of precipitation-hardened steels (15-5PH and 17-4PH) passivated in citric acid. The electrochemical techniques used were electrochemical noise and potentiodynamic polarization; the results showed that the passivation films formed with citric acid performed very similarly to the passive films formed using nitric acid.

Other authors such as Bragaglia et al. [26] and Marcelino et al. [27] used potentiodynamic polarization and electrochemical impedance spectroscopy to characterize the oxide film through charge transfer processes in passivated 304 austenitic stainless steel. El-Taib Heakal et al. conducted a study on the electrochemical characteristics of stainless steels while varying the pH concentrations in aerated solutions using PP and EIS. The results indicated that a decrease in the pH of the solution promotes the formation of oxide films, which improves corrosion resistance [28,29]. Precipitation-hardened stainless steels offer not only corrosion resistance but also high mechanical strength and ease of heat treatment, which means their research involves different approaches [30]. A study that evaluated the presence of pitting over a 13-year storage period prompted a failure analysis on AM350 steel bellows in a marine atmosphere. Additionally, other researchers have studied the low-cycle fatigue behavior of AM355 and precipitation-hardened martensitic stainless steels at different temperatures [31,32,33,34,35]. Comparative machining studies were conducted on the Custom 450 alloy, employing carbide tools that were coated with TiCN and TiAl [36,37]. Custom 450 and AM350 stainless steels have been the subject of corrosion evaluation studies in the aeronautical sector, constituting one of the most significant applications of PHSS [38,39]. Studies on the corrosion behavior of passivated PHSS steels such as CUSTOM450 and AM350 for use in the aeronautical sector were carried out by Samaniego et al. [40]. They used electrochemical noise and electrochemical impedance spectroscopy to study the corrosion kinetics of the passivation film in acid immersions. Their study showed that CUSTOM 450 PHSS martensitic steel showed the greatest performance.

In this work, we aimed to study the effectiveness of passivation treatments on 17-7PH stainless-steel samples. The treatments were performed with citric and nitric acid solutions maintained at 49 °C. Different immersion times of 90 min were used, and subsequent exposure in 3.5 wt.% NaCl solution was employed, along with the application of cyclic potentiodynamic polarization (CPP). A microstructural analysis was performed using scanning electron microscopy (SEM), and characterization of the passive layer was performed using X-ray photoelectron spectroscopy (XPS).

## 2. Materials and Methods

### 2.1. Materials

The commercial stainless steels employed were of the 17-7PH (Aerospace Material Specifications, AMS 5528) and 410 SS (AMS 5613) varieties and obtained from sheets with dimensions of 304.8 × 304.8 mm and a thickness of 0.81 mm. Also, a 410 SS sheet with dimensions of 152.4 × 152.4 mm and a thickness of 2.28 mm was used. The chemical compositions of 17-7PH and 410 SS, as provided by the supplier (McMaster-Carr, Elmhurst, IL, USA) on their datasheet, are displayed in Table 1. Using the metallography technique, 17-7PH and 410 SS samples were prepared: they were ground and polished with grade 400, 500, 600, and 800 SiC sandpaper and cleaned for 10 min using ultrasound in ethanol and deionized water [40].

### 2.2. Passivation Treatment

The samples underwent passivation according to the procedures outlined in the ASTM A967-09 standard [17] and in earlier investigations into the estimation of passivation parameters.

1. The samples were cleaned ultrasonically according to ASTM A380-17 [41]. This cleaning process involved immersing the samples in ethanol for a duration of 10 min; after that, they were rinsed with deionized water to remove any residual contaminants. Ultrasonic cleaning is a highly effective method for removing any impurities that could compromise subsequent treatments [42].
2. Two passivation solutions were prepared for the treatment of the samples. The first solution consisted of citric acid (C_6_H_8_O_7_) at a concentration of 15 wt.% and a pH of 1.51. The second solution comprised nitric acid (HNO_3_) at a concentration of 20 *v*/*v*% and a pH of 0.41.
3. After preconditioning the passivating solutions at 49 °C, the samples were carefully submerged. The immersion time was 90 min, ensuring adequate exposure to the passivation solution for effective treatment.
4. Finally, rinsing in deionized water and air drying were carried out.

**Figure 1:**
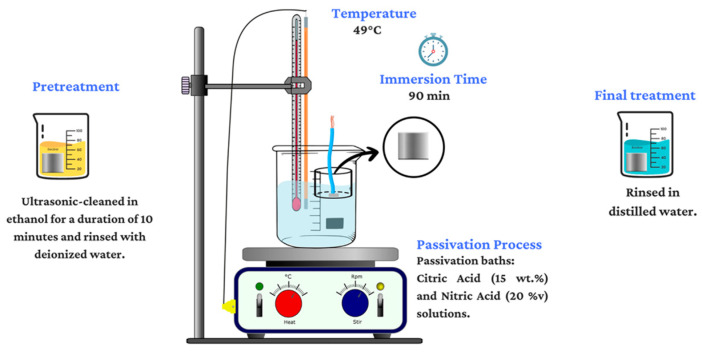
Diagram of passivation treatment of 17-7PH and 410 SS steels.

The passivation process described above is illustrated in Figure 1, while a description of the samples is provided in Table 2.

### 2.3. SEM Microstructural Characterization

Scanning electron microscopy (SEM, JEOL-JSM-5610LV, Tokyo, Japan) was used to determine the microstructures of 17-7PH and 410 stainless-steel samples in initial conditions and after corrosion tests (following testing). Analyses were performed secondary (SE) and backscattered electron (BE) detectors at 40×, 70×, 200×, and 500× magnification, operating at 15 and 20 kV (WD = 8.5 mm).

### 2.4. Electrochemical Testing

**Figure 2:**
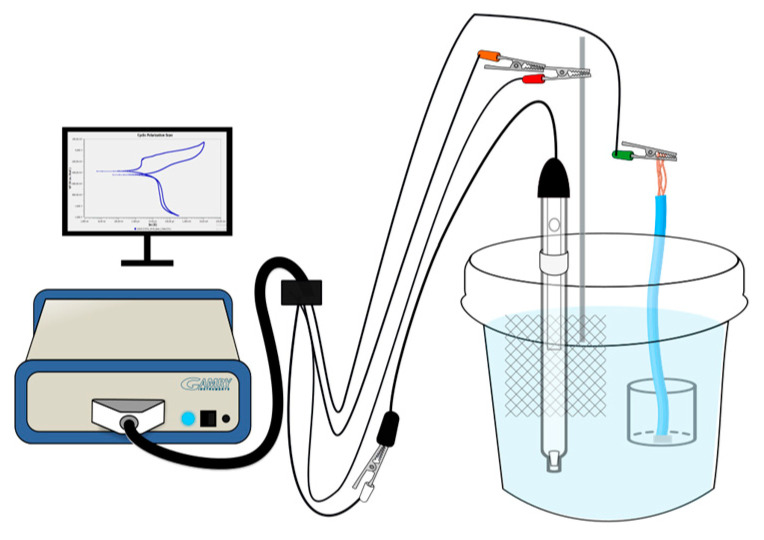
Conventional three-electrode cell configuration used in the CPP tests.

An experiment was conducted to investigate the interfacial characteristics of the 17-7PH stainless-steel samples, including active–passive behavior and susceptibility in a corrosive environment. This included immersing the samples in a 3.5 wt.% sodium chloride solution at ambient temperature [43,44,45,46]. The aim was to replicate the chemical and chloride conditions found in marine environments, which are known to cause localized corrosion and pitting. The equipment used for the testing was an Interface 1000 potentiostat/galvanostat/ZRA (Gamry Instruments, Warminster, PA, USA) with an exposed sample area of 1.0 cm^2^ along with a standard electrochemical cell (illustrated in Figure 2) consisting of three electrodes. The passivated stainless-steel samples (17-7PH and 410 SS) were used as the working electrode (WE). The reference electrode (RE) employed was a saturated calomel electrode (SCE), and the counter electrode (CE) was a platinum mesh. Tests were conducted in duplicate.

Initial observations of the electrochemical corrosion potential (E_corr_) were made until it reached a stable value. Cyclic potentiodynamic polarization (CPP) testing was conducted in accordance with the ASTM G61-86 standard [44] to obtain data regarding the kinetics of the dissolution reaction in electrochemical corrosion. CPP curves were obtained, and a potential scan was carried out from −1 V to +1.2 V with respect to E_corr_ at a scan rate of 1 mV/s [46,47]. Prior to testing, a one-hour open-circuit potential (OCP) was applied to the passivated sample (working electrode).

### 2.5. X-ray Photoelectron Spectroscopy (XPS) Characterization

The chemical compositions of the oxide layer surface and valence states of 17-7PH and 410 SS were determined through high-resolution XPS analysis. This analysis was conducted using a Thermo Fisher Scientific ESCALAB 250 Xi instrument from Waltham, MA, USA. The equipment was operated at a pressure of 10 mBar. The evaluation conditions included high-resolution zones and an analysis radius of µm. The step energy, defined in electron volts (eV), was determined with a step size of 0.1 eV, and the “take-off angle” was set to 45°. The photoelectrons were excited and measured with a monochromatic Al KAlpha X-ray source with 1486 eV of energy.

## 3. Results and Discussion

### 3.1. Scanning Electron Microscopy (SEM) and Microstructural Analysis

**Figure 3:**
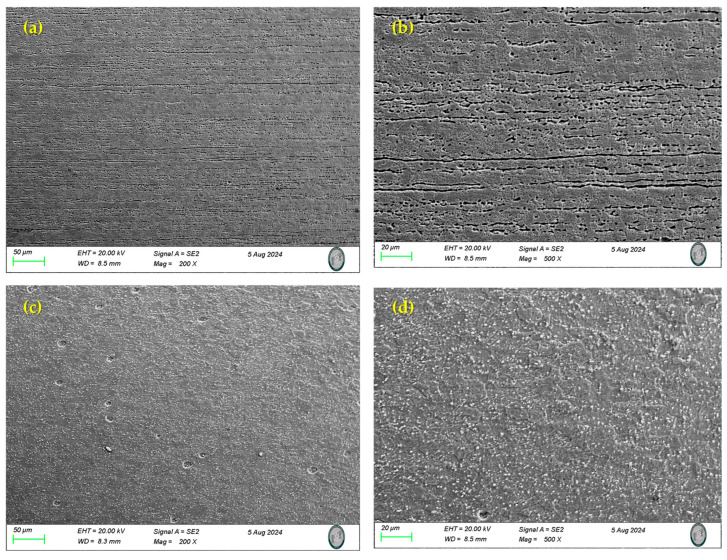
SEM-SE Microstructure of martensitic of samples (initial conditions): (**a**,**b**) 17-7PH, (**c**,**d**) 410 S.

The SEM technique was used to study the microstructures of the samples in their as-received conditions. Figure 3 shows the microstructures of the stainless steel under study in the annealed condition. 17-7PH (Figure 3a,b) stainless steel is semi-austenitic and exhibits a delta ferrite (δ) and austenite (γ) phase microstructure, and the microstructure of 410 SS (Figure 3c,d) consist of austenite (γ), a delta (δ) ferrite phase, and chromium carbides respectively. In stainless steels, austenite (γ) is a thermodynamically stable phase, and this phase can be transformed via a hardening heat treatment [48,49,50]. Martensitic stainless steels have a higher carbon content which reduces their corrosion resistance but enhances mechanical properties such as toughness. However, this also makes them more susceptible to chromium carbide precipitation at grain boundaries.

### 3.2. Pitting Resistance Equivalent Number (PREN)

The precipitation-hardening stainless-steel alloy 17-7PH is semi-austenitic, being austenitic in the annealed condition and martensitic in the hardened condition, and 410 SS is of the martensitic type. 

Localized corrosion of stainless steel can be measured using the pitting resistance equivalent number (PREN). Using theoretical foundations and based on their chemical composition, the resistance to pitting corrosion of different types of stainless steel can be estimated [51,52]. High PREN values indicate increased corrosion resistance. It is important to note that the content of alloying elements such as chromium, molybdenum, and nitrogen determines the PREN (see Equation (1)). The PREN results indicate that 17-7PH steel has better resistance to pitting corrosion than 410 SS (Table 3).

PREN = %Cr + 3.3 (%Mo + 0.5%W) + 16%N (1)

### 3.3. XPS Characterization

**Figure 4:**
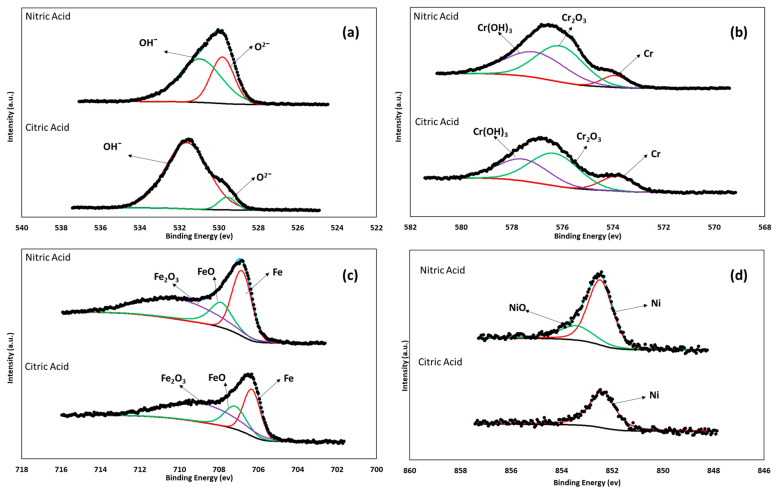
X-ray photoelectron spectra and peak binding energy for 17-7PH stainless steel passivated in nitric and citric acids at 49 °C for 90 min: (**a**) O 1s, (**b**) Cr 2p^3/2^, (**c**) Fe 2p^3/2^, and (**d**) Ni 2p^3/2^.

**Figure 5:**
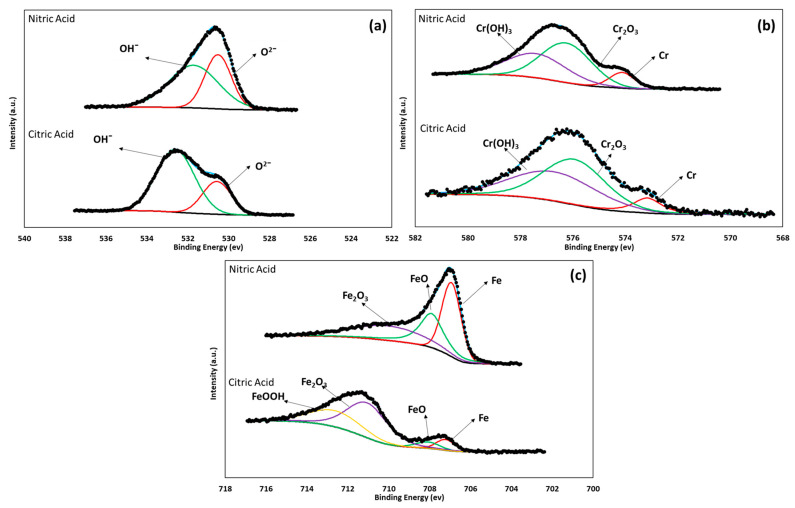
X-ray photoelectron spectra and peak binding energy for 410 SS passivated in nitric and citric acids at 49 °C for 90 min: (**a**) O 1s, (**b**) Cr 2p^3/2^, (**c**) Fe 2p^3/2^.

The surface chemicals, valence states, and oxide layer compositions on the passivated steels were determined via XPS measurements. The NIST database (National Institute of Standards and Technology; Gaithersburg, MD, USA) was employed to conduct deconvolution calculations of the XPS spectra and identify the chemical compounds through their corresponding binding-energy peaks. XPS analysis was performed using Avantage software (Waltham, MA, USA). The high-resolution XPS spectra are shown in Figure 4 and Figure 5, obtained for 17-7PH and 410 SS steels passivated in citric and nitric acid. For 17-7PH the spectra measured were O1s, Cr2p^3/2^, Fe2p^3/2,^ and Ni 2p^3/2^, and for 410 SS, the spectra only consisted of O1s, Cr2p^3/2^, and Fe2p^3/2^ due to the low concentration of Ni in the alloy. Both 17-7PH and 410 SS displayed four main chemical species in the passive film: Cr_2_O_3_, FeO, Fe_2_O_3_, and Cr (OH)_3_. Along with the increased concentration of Ni in the 17-7HP samples, NiO was also detected through XPS. The presence of oxides gives rise to chromium hydroxides, and the formation of oxides on the surface due to OH^−^ was reported by Natajaran et al. [52]. The binding energy peaks obtained for FeO and Fe_2_O_3_ ranged from 706 eV to 713.2 eV, respectively.

Figure 4b and Figure 5b show the Cr 2p^3/2^ spectra for 17-7PH and 410 SS, respectively. It was observed that there was a main peak around 576 eV, which is attributable to the enrichment of Cr_2_O_3_ from the passivation process [53,54]. In Figure 3c and Figure 4c, the analysis of the O 1s and Fe 2p^3/2^ contributions in most of the deconvolutions shows different iron oxides and hydroxides, mainly linked to Fe^3+^. According to Jung and Mesquita [55,56], there is a correlation between the ratio of Fe to Cr species in the passive film and the formation conditions. Table 4 shows the binding energies and relative amounts of each component in the passive film. It can be seen from Table 4 that the predominant chemical species was Cr_2_O_3_, and this was because the steel was passivized via immersion in citric and nitric acid. For environments where Fe is expected to be selectively dissolved, there would be an enrichment of Cr species in the formed passive film, possibly leading to increased protectiveness of the formed film. Previous studies have shown that passive films have a bilayer structure [57,58,59]. The inner layer is compact, continuous, and composed mainly of chromium (III) oxide, whereas the outer layer is porous and generally composed of iron and chromium oxides and hydroxides, respectively.

### 3.4. Electrochemical Measurements

**Figure 6:**
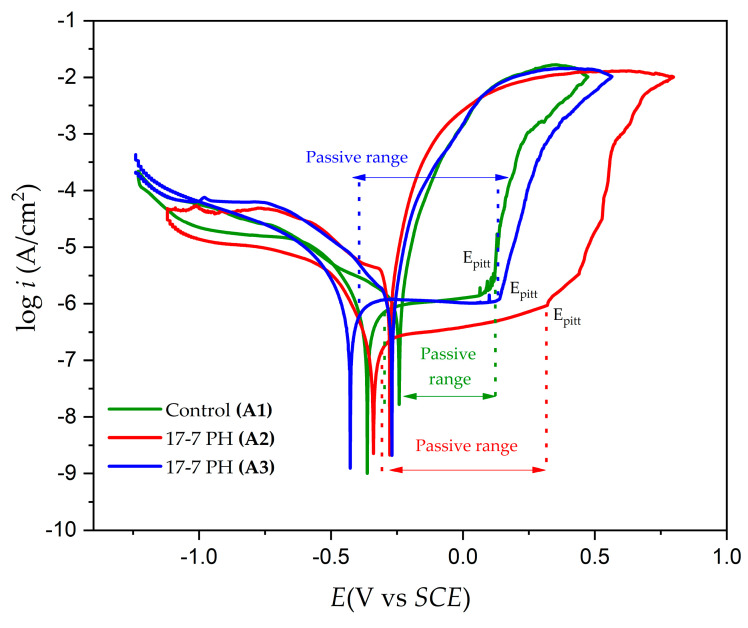
Cyclic potentiodynamic polarization curves of 17-7PH samples passivated in citric acid and nitric acid at 49 °C for 90 min after exposure to NaCl solutions.

**Figure 7:**
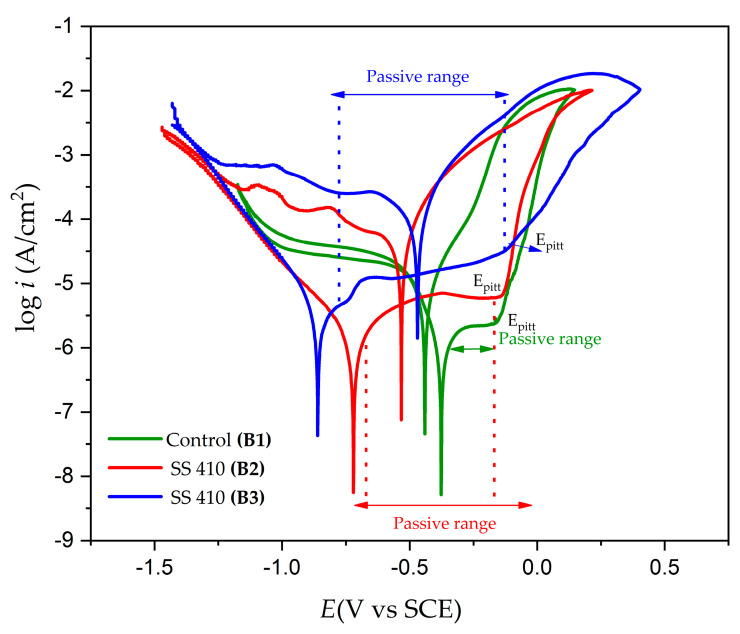
Cyclic potentiodynamic polarization curves of 410 SS samples passivated in citric acid and nitric acid at 49 °C for 90 min after exposure to NaCl solutions.

Corrosion kinetics trends can be observed from the cathodic and anodic regions of the cyclic potentiodynamic polarization curves. Figure 6 and Figure 7 show the CPP obtained for the 17-7PH and 410 SSs, respectively, passivated in acid baths at 49 °C for 90 min and immersed in 3.5 wt.% NaCl solution. Table 5 shows the electrochemical parameters obtained using CPP curves for both 17-7PH and 410. In the CPP curves, the Tafel extrapolation technique was used to determine the corrosion current density, i_corr_ (µA·cm^−2^); the corrosion potential, E_corr_ (V); and the corrosion rate [5,60,61,62,63,64,65]. The corrosion current density was determined at a range of ± 300 mV in the linear section of the potentiodynamic polarization curves in at least one period of current using Tafel extrapolation [61,62,63,64,65].

The CPP curves in Figure 6 and Figure 7 indicate that all the systems’ anodic and cathodic reactions showed a mixed activation, followed by passivation. For both materials, the samples that were subjected to both nitric and citric acid passivation solutions displayed larger passivation ranges and similar magnitudes of $i_{corr}$ and CR. The passivation ranges for the samples in citric acid were 0.646 V for the 17-7PH sample and 0.450 V for the 410 SS sample, while for the samples in nitric acid, the passivation ranges were 0.857 V for the 17-7PH sample and 0.427 V for the 410 SS sample. The general corrosion rates for all the samples exposed to the solution were around 10^−2^–10^−3^ mm/yr, showing that all the samples displayed low corrosion rates due to the passive films formed on them. There were higher corrosion rate values in the 410 SS sample submerged in 3.5 wt.% NaCl, most likely due to its lower concentration of Cr, Ni, and Mo compared to 17-7PH steels. According to the CPP plots, all the samples exhibited positive hysteresis, which indicates that they are susceptible to localized corrosion. For both steels, the passive current values increase for the samples exposed to nitric acid relative to the passive current measured on the control samples. The results for the 17-7PH and 410 SSs passivated and immersed in NaCl solution show that the *E_corr_* value is more noble following passivation with citric acid, with values of −0.341 for sample A2 and −0.687 sample B2, while the samples in nitric acid had more active values, ranging from −0.420 V for A3 to −0.858 for B3, respectively. Figure 6 and Figure 7 show the results of the pitting potentials (*E_pit_*) of the steels passivated in citric acid (0.311 V sample A2 and −0.154 sample B2) and in nitric acid (0.133 V sample A3 and −0.183 sample B3). Relative to the control samples, the pitting potentials of the passivated 17-7PH showed a marked increase in values, while for 410 SS, the samples stayed relatively close to the $E_{pit}$ of the control samples.

The cyclic potentiodynamic polarization curves of the 17-7PH and 410 SS steels (Figure 5 and Figure 6) present very similar trends, showing that there was passivation in the anodic reaction but a difference between its pitting potential due to the different electrochemical reactions in the NaCl solution. The formation of a passivation layer on the surface of stainless steel determines its corrosion resistance and activates this passivation protection mechanism in Fe-Cr alloys. Chromium oxides play an important role in passive films and anodic ^−^OH interactions in terms of developing the protective mechanism. This protective process creates a passive layer of Cr-rich oxides and oxyhydroxides that do not allow oxygen into the inner layer and protects the base material from corrosive ions such as Cl^−^ when stainless-steel samples are exposed [66,67,68]. According to the CPP results, both materials that were passivated using nitric or citric acid showed larger passive ranges and increased pitting potential compared to the control samples. This increase in protectiveness is most likely due to the enrichment of Cr species in the formed passive films. This can be seen in the XPS results, where the dominant species in the passive film was Cr_2_O_3_.

Only in sample B3 of the 410 SSs passivated and immersed in a 3.5% NaCl solution did pseudo-passivation occur. Passive films created on martensitic steels may be in a semi-stable condition due to rising current density with an increase in anodic potential instead of reaching the stable state within the passive region. This fluctuation is caused mainly by chloride ions (Cl^−^), which are adsorbed onto the steel surfaces and can diffuse through holes in the passive film [69,70,71,72,73]. The development of the Cr (OH)_3_ film may be related to the pseudo-passivation phenomenon observed in the cyclic potentiodynamic polarization curves of the passivated B3 sample. In previous works, Cr (OH)_3_ it was reported that may inhibit the iron dissolution process, isolate corrosive electrolytes, and lower the number of active sites in the iron dissolution process of stainless steel [74,75,76,77]. Several authors indicate that [67,68,69,70,71] citric acid can be a sustainable alternative to nitric acid. In future works, it is important to adjust some parameters of the passivation process, such as increasing the passivation duration of citric acid solutions due to the characteristics of this electrolyte.

**Figure 8:**
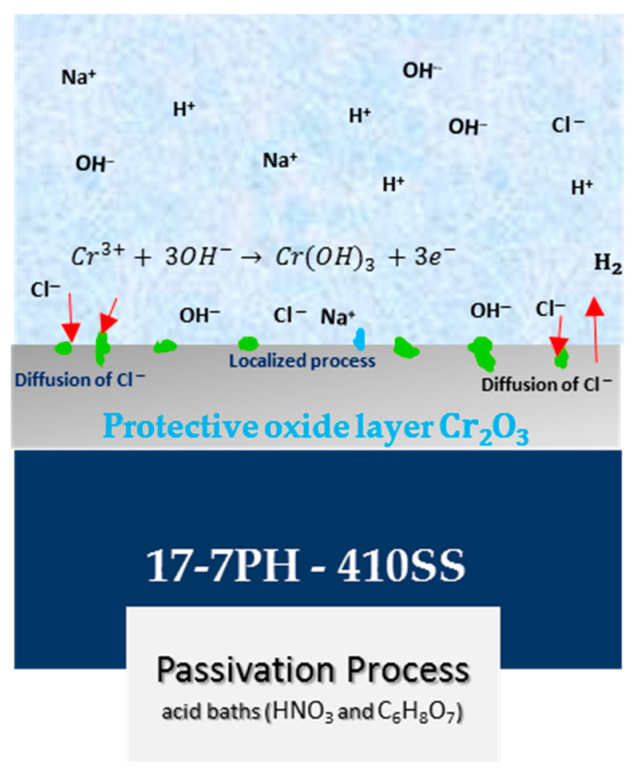
Diagram of the protectiveness of the formed passive film in citric and nitric acid baths for 17-7PH and 410 SS steels exposed to 3.5 wt.% NaCl solution.

Iron oxide (formed via anodic dissolution of Fe) and chromium oxide films, which are frequently found on stainless steels, form in the passive layer [78,79,80,81,82,83]. The protectiveness of the formed passive film (see Figure 8) is most likely due to the greater enrichment of chromium oxide and hydroxide species in the passive film compared to that in air-formed passive films. The enrichment of the passive film is due to the passivation processes, where the surface contaminates are removed and oxidation of the surface allows the formation of protective film. This enriched passive film then prevents aggressive species such as O and Cl^−^ from penetrating the base metal, delaying the onset of corrosion [84].

### 3.5. SEM Surface Characterization

Surface characterization was carried out via SEM using backscattered electron (BSE) imaging. The morphology of the surfaces of the stainless-steel samples was determined, and then the samples were tested in 3.5 wt. % NaCl. Semiquantitative elemental compositions (concerning the elements present in the analyzed areas) were measured using EDS.

**Figure 9:**
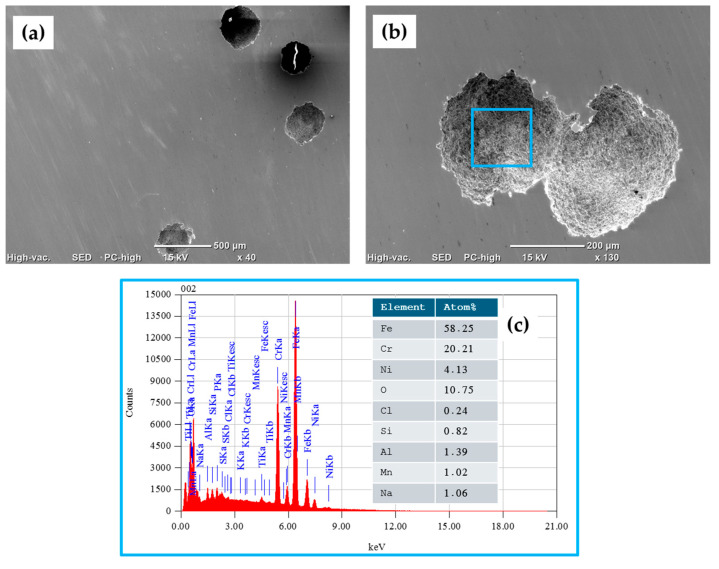
SEM-BSE surface morphology of 17-7PH stainless-steel sample passivated in citric acid after CPP test in 3.5 wt.% NaCl electrolyte solution: (**a**) 40× magnification, (**b**) 130× magnification, and (**c**) EDS spectrum and elemental analysis percentages.

Figure 9a–c show the morphology of the surfaces, as analyzed via SEM-BSE, of the 17-7PH stainless steel corresponding to different magnifications of the samples passivated in citric acid. The samples had a pit density measuring 250–600 µm when they were passivated in citric acid. As shown in the EDS spectrum in Figure 9c, obtained from Figure 9b (blue box), the elemental analysis identified the presence of alloy elements such as iron, chromium, nickel, silicon, and manganese, among others, as well as the elements of the corrosive agent: chlorine and sodium.

**Figure 10:**
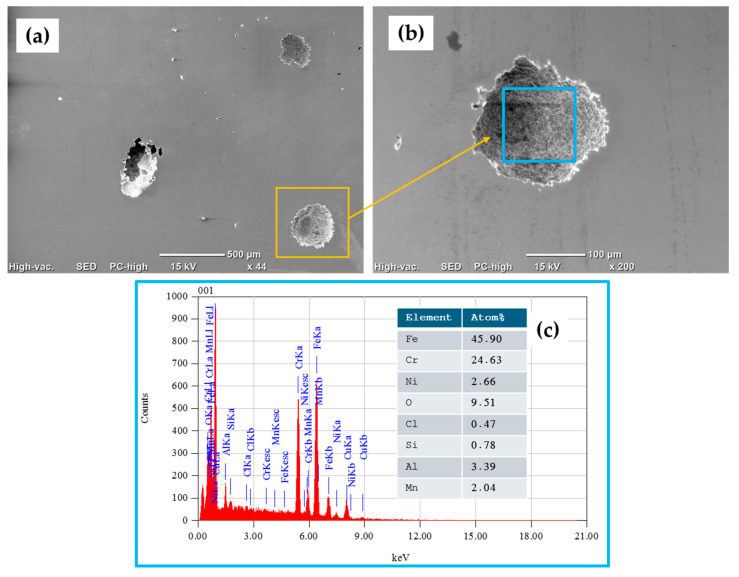
SEM-BSE surface morphology of 17-7PH stainless-steel sample passivated in nitric acid after CPP test in 3.5 wt.% NaCl electrolyte solution: (**a**) 40× magnification, (**b**) 130× magnification, and (**c**) EDS spectrum and elemental analysis percentages.

Figure 10a–c show SEM-BSE images of the morphology of the surface of the 17-7PH stainless steel corresponding to different magnifications of the samples passivated in nitric acid. The morphologies show some pitting caused by the sodium chloride solution for the samples passivated in nitric acid, where the pits range in size from 100 to 300 µm. In the EDS spectrum in Figure 10c, obtained from Figure 10b (blue box), the elemental analysis identified the presence of alloy elements such as iron, chromium, nickel, silicon, and manganese, among others, as well as the elements of the corrosive agent: chlorine and sodium.

**Figure 11:**
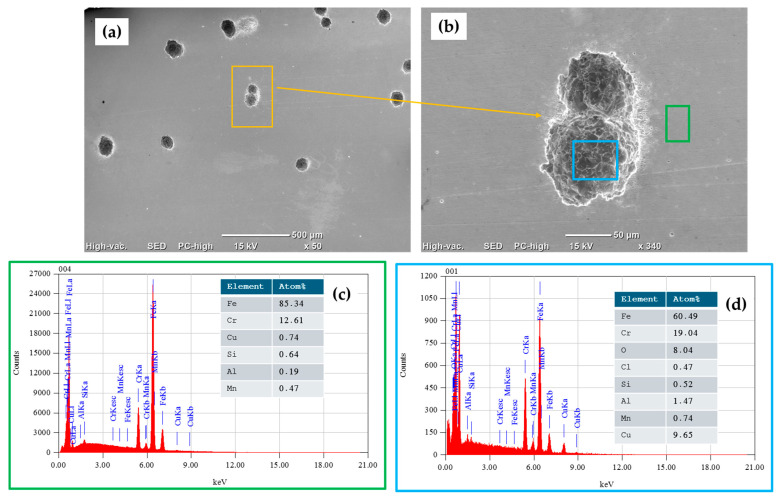
SEM-BSE surface morphology of 410 SS sample passivated in citric acid after CPP test in 3.5 wt.% NaCl electrolyte solution: (**a**) 50× magnification, (**b**) 340× magnification, and (**c**,**d**) EDS spectrums and elemental analysis percentages.

Figure 11a–c shows SEM-BSE images of the morphology of the surface of the 410 SS corresponding to different magnifications of the samples passivated in citric acid. The samples had a considerable density of pits measuring 50–100 µm when they were passivated in citric acid. As shown in the EDS spectra in Figure 11c,d, obtained from Figure 11a (orange box) and Figure 11b (blue and green box), elemental analysis identified the presence of alloying elements such as iron, chromium, copper, silicon, and manganese, among others, in an area without pits (green box), and in the area with pits, there were elements such as iron and chromium that decreased in quantity.

**Figure 12:**
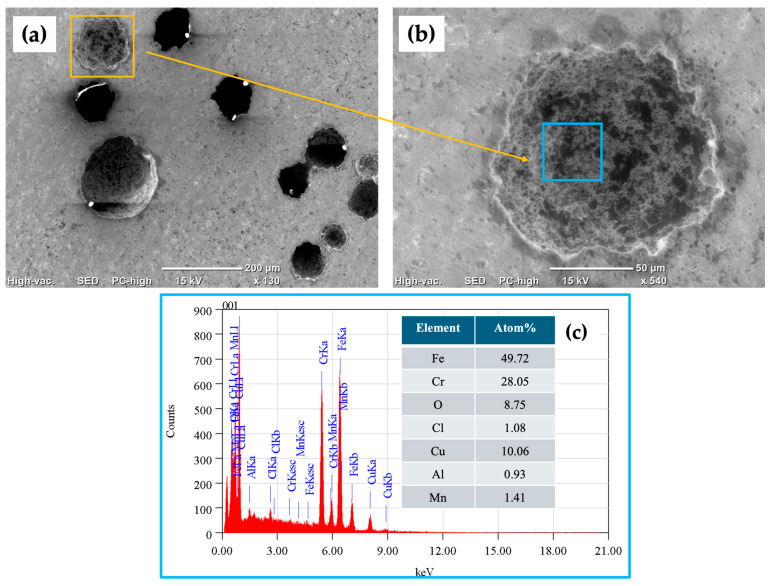
SEM-BSE images of surface morphology of 410 SS sample passivated in nitric acid after CPP test in 3.5 wt.% NaCl electrolyte solution: (**a**) 130× magnification, (**b**) 540× magnification, and (**c**) EDS spectrum and elemental analysis percentages.

Figure 12a–c show SEM-BSE images of the morphology of the surface of the 410 SS corresponding to different magnifications of the samples passivated in nitric acid. The morphology shows some pitting caused by the sodium chloride solution for the samples passivated in nitric acid, where the pits have a size ranging from 100 to 200 µm. As shown in the EDS spectrum in Figure 12c, obtained from Figure 12b (blue box), the elemental analysis identified the presence of alloy elements such as iron, chromium, copper, and manganese, among others, as well as the elements of the corrosive agent, chlorine and sodium.

The passive film on stainless steel has a double-layer structure, with each layer being rich in Fe and Cr, respectively. Chromium oxides play a crucial role in the corrosion resistance of stainless steel. Cr^3+^ exhibits higher corrosion stability than FeO and Fe_2_O_3_ oxides. Thus, the Cr_2_O_3_ content in the passive film of stainless steel is a main factor determining the stability and anti-corrosion properties of the steel itself. Defects will form at the substrate and the passive film interface, leading to localized corrosion nucleation (generating pitting). It is important to note that the defect density of the iron-rich outer layer is higher than that of the chromium-rich inner layer. This difference in defect density could cause the passive film of stainless steel to absorb a significant amount of Cl^−^.

## 4. Conclusions

This research investigated the passive state of 17-7PH and 410 SSs immersed in citric and nitric acid baths at 49 °C for 90 min and immersed in 3.5 wt.% NaCl solutions. Considering the results of the experiments conducted, the following can be concluded:

- SEM characterization under the initial conditions and conducted on the samples in an as-received state indicated that the 17-7PH presented a microstructure with a martensitic (α′) phase, while 410 SS contained a microstructure consisting of austenite (γ), a delta (δ) ferrite phase, and chromium carbides.
- The PREN results indicate that 17-7PH (18.92) steel has better resistance to pitting corrosion than 410 SS (13.5).
- According to the cyclic potentiodynamic polarization results, 17-7PH steel passivated in citric acid exhibited lower corrosion rate values (in the order of ×10^−3^ mm/yr).
- The application of nitric acid passivation caused the surface to become susceptible to localized corrosion.
- Passivation of 410 SS in acid nitric showed a trend in the cyclic potentiodynamic polarization curves, although it was not fully defined.
- The passivated 17-7PH and 410 SS steels exhibited positive hysteresis, which indicates that they are susceptible to localized corrosion.
- The results obtained following SEM analysis of the electrochemically tested samples indicated the presence of localized corrosion (pitting), and for the 410 SS, a higher density of pits compared to that for 17-7PH stainless steel was found, showing 50 and 100 μm pit sizes, respectively.
- The XPS analysis indicated different chemical species on the surface films of the 17-7PH and 410 SSs, such as Cr_2_O_3_, Cr (OH)_3_, FeOOH, and Fe_2_O_3_. The passive films contained iron chromium oxide and hydroxide.
- The 17-7PH SS samples passivated at 49 °C for 90 min in citric and nitric baths exhibited the best performance.
- The application of the citric acid passivation process to passivated stainless steels could be an environmentally friendly alternative to the frequently used nitric acid passivation process.
- The potentiodynamic polarization results indicated that 17-7PH stainless steel passivated in citric and nitric acid showed lower corrosion rate values (in the order of ×10^−3^ mm/yr).
- XPS analysis allowed us to determine that the surface film of the 17-7PH and 410 SS samples analyzed in this work consisted of different chemical species, such as Cr_2_O_3_ and Fe (OH)O.

## Figures and Tables

**Table 1:** Chemical composition of 17-7PH and 401 stainless steels (wt.%).

Material	Elements (wt.%)												
	C	Cr	Ni	Mo	Mn	Cu	Ti	N	Nb	W	V	Al	Fe
17-7PH	0.09	16.64	7.17	0.19	0.88	0.24	0.10	0.10	0.034	0.034	0.106	1.1	Balance
410 SS	0.15	13.5	0.75	--	0.5	--	--	--	--	--	--	0.5	Balance

**Table 2:** Nomenclature regarding the 17-7PH and 410 stainless-steel samples.

Sample	Material	Temperature (°C)	Time (min)	Passivation Baths
A1 (Control)	17-7PH	--	--	--
B1 (Control)	410 SS	--	--	--
A2	17-7PH	49	90	Citric Acid C_6_H_8_O_7_
B2	410 SS	49	90	
A3	17-7PH	49	90	Nitric Acid HNO_3_
B3	410 SS	49	90	

**Table 3:** Pitting resistance equivalent numbers of the 17-7PH and 410 SS samples.

Sample	Cr	Mo	N	W	PREN
17-7PH	16.64	0.190	0.10	0.034	18.92
410SS	13.5	-	-	-	13.5

**Table 4:** Relative amounts of Cr, Cr_2_O_3_, and Cr (OH)_3_ and Fe, FeO, Fe_2_O_3_, and FeOOH in the formed passive film.

Samples:			17-7		410 SS	
Element	Peak	Binding Energy (eV)	Citric	Nitric	Citric	Nitic
Fe 2p^3/2^	Fe metal	706.6	0.296	0.345	0.071	0.395
	FeO	708.4	0.207	0.214	0.054	0.307
	Fe_3_O_4_	709.8	0.497	0.441	0.573	0.298
	FeOOH	710.3	-	-	0.302	-
Cr 2p^3/2^	Cr metal	574.2	0.154	0.096	0.094	0.107
	Cr_2_O_3_	576.1	0.497	0.526	0.499	0.501
	Cr (OH)_3_	577.3	0.349	0.378	0.407	0.392

**Table 5:** Parameters obtained via CPP for passivated 17-7PH and 410 SSs evaluated in 3.5 wt.% NaCl solution.

Sample	E_corr_ (Volts)	E_pit_ (Volts)	E_rp_ (Volts)	i_corr_ (A/cm^2^)	σ_icorr_ (A/cm^2^)	i_pass_ (A/cm^2^)	Passive Range (Volts)	CR (mm/year)	Hysteresis
Control A1	−0.364	0.062	−0.238	1.146 × 10^−6^	±4.137 × 10^−3^	1.023 × 10^−6^	0.323	1.325 × 10^−2^	Positive
A2	−0.341	0.311	−0.276	1.8638 × 10^−7^	±4.292 × 10^−3^	4.4791 × 10^−7^	0.450	2.1345 × 10^−3^	Positive
A3	−0.420	0.133	−0.263	2.5118 × 10^−7^	±4.184 × 10^−3^	1.1230 × 10^−6^	0.427	2.9131 × 10^−3^	Positive
Control B1	−0.380	−0.170	−0.440	1.113 × 10^−6^	±2.650 × 10^−3^	2.1682 × 10^−6^	0.492	1.2908 × 10^−2^	Positive
B2	−0.687	−0.154	−0.531	1.1945 × 10^−6^	±2.361 × 10^−3^	5.7451 × 10^−6^	0.646	1.3854 × 10^−2^	Positive
B3	−0.858	−0.183	−0.468	2.5971 × 10^−6^	±2.636 × 10^−3^	1.5502 × 10^−5^	0.857	3.0121 × 10^−2^	Positive

## Data Availability

The original contributions presented in the study are included in the article, further inquiries can be directed to the corresponding authors.
